# Salt Bridge in Aqueous Solution: Strong Structural Motifs but Weak Enthalpic Effect

**DOI:** 10.1038/s41598-018-31935-z

**Published:** 2018-09-11

**Authors:** Svetlana Pylaeva, Martin Brehm, Daniel Sebastiani

**Affiliations:** Martin Luther University Halle–Wittenberg, Institute of Physical Chemistry, 06120 Halle, Saale Germany

## Abstract

Salt bridges are elementary motifs of protein secondary and tertiary structure and are commonly associated with structural driving force that increases stability. Often found on the interface to the solvent, they are highly susceptible to solvent–solute interactions, primarily with water but also with other cosolvents (especially ions). We have investigated the interplay of an Arginine–Aspartic acid salt bridge with simple salt ions in aqueous solution by means of molecular dynamics simulations. Besides structural and dynamical features at equilibrium, we have computed the mean force along the dissociation pathway of the salt bridge. We demonstrate that solvated ions influence the behavior of the salt bridge in a very specific and local way, namely the formation of tight ionic pairs Li^+^/Na^+^–Asp^−^. Moreover, our findings show that the enthalpic relevance of the salt bridge is minor, regardless of the presence of solvated ions.

## Introduction

Salt bridges are interactions of amino acids with opposite charge where at least two heavy atoms lie within a hydrogen bonding distance^1,2^. Often found in solvent exposed parts of proteins, they are susceptible to external interactions, primarily with water. The Coulomb attraction of opposite charges is then screened by polar water molecules, and an outcome ranges from a contact ion pair to separately solvated ionic groups. This indicates that the relative strength of a salt bridge usually assumed to be around a couple of k_*B*_T and strongly depends on the environment^1,3,4^. The Lysine-Glutamine salt bridge was investigated computationally in vacuum and in water in an attempt to model hydrophobic environment in protein core versus solvent accessible position^5^. It was shown that in a hydrophobic environment, the peptide is in a molecular form^3^, while upon addition of water molecules it switches to zwitterionic form, forming a salt bridge. However, the salt bridge is significantly weakened upon further hydration.

If one considers a realistic environment of proteins in a living cell, it contains not only water but different co-solvents, ions. And free ions in solution can further influence salt bridges by extra shielding of charges (unspecific influence), and competing amino-acid–free ion pair formation (specific influence)^6–13^.

A lot of research effort, both at the experimental and computational level, has been devoted to the understanding of the solvation processes and structures of large and small ions, the question of ion pairing and the indirect structural effects leading to effective interactions between proteins and dissolved salts. The most prominent topic in this context are the ‘salting in’ and ‘salting out’ phenomena that led to the Hoffmeister series of ions and their kosmotropic/chaotropic characters^14^. The commonly accepted causality chain was that the presence of ions changes the water structure which induces a change in the ability of water to interact with the protein surface. The corresponding terms kosmotropic and chaotropic indicate that a specific ion can make the hydrogen bond network more rigid–kosmotropic or more flexible–chaotropic. An interesting related ongoing debate is whether the properties of solvating water molecules determine the behavior of proteins or whether the properties of the protein surface determine the behavior of the surrounding water (‘enslaved water’ versus ‘enslaving water’)^15–17^.

Ion specific influence on water stucture was observed experimentally by means of NMR measurements and computationally on different levels of theory^18–24^; the effect was shown to go beyond the first solvation shell, i.e. beyond water molecules in direct contact with ions. Authors^25,26^ described to which extent water dynamics depended on specific ions and their concentrations: at low salt concentration ion specific effects are present–either slowing down or speeding up water motion, at high salt concentrations the effect is unspecific due to increased viscosity–water dynamics is slowed down. Recently, some authors argued that specific influence of Hofmeister ions is dominant^27^: only properties of species directly interacting with ions change, whereas properties of bulk solvent stay completely intact (see Zhang *et al*.^28^ and papers cited therein).

In this work, we investigate a common salt bridge and quantify the effect of a series of solvated ions on both structure and dynamics as well as the consequent influence on thermodynamic properties. Do simple ions influence the salt bridge? Is this influence specific or unspecific? We have performed MD simulations of the Arginine-Aspartic acid dimer in aqueous solution, in a setup similar to the one chosen by Chong *et al*.^29^. While being relatively simplistic, this model allowes to enhance our focus on the interplay of water–ion–amino acid interactions, excluding other more subtle interactions in a protein. It is known that MD simulations are highly succeptible to minute changes in force fields^29–31^. Interactions with ions require additional care to be able to account for polarization effects. This can be achieved either by employing polarizable force fields^32^ or scaling ion parameters^33–35^ to ensure that the results of the computer simulations reproduce specific experimental findings. For our study, we have chosen the latter option; we use Kirkwood-Buff-type force field ion parameters proposed for non-polarizable force fileds by Gee *et al*.^35^ together with force field parameters and water model relying on a benchmark study of the salt bridge by Chong *et al*.^29^.

## Results

**Figure 1:**
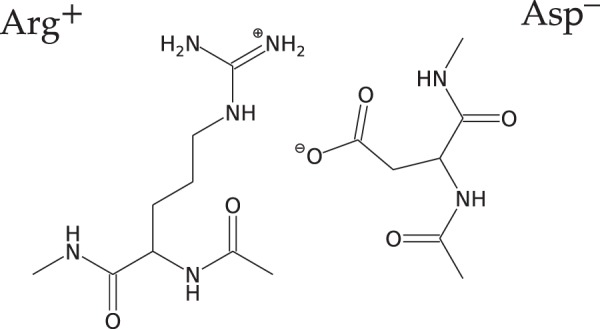
Structure of the studied salt bridge.

We have investigated the effect of several ions from the Hofmeister series on the stability of a capped Arginine-Aspartic acid salt bridge (Fig. 1) in pure water and in 1 M aqueous solutions of NaCl, NaI, LiCl, and LiI. We have performed Umbrella pulling classical molecular dynamics simulations of those systems under ambient conditions (see Methods section for more details).

### Water rotational dynamics

**Figure 2:**
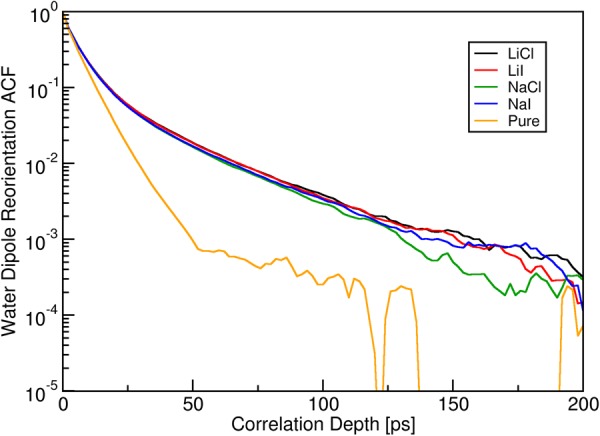
Vector autocorrelation functions of the dipole vector of water for all studied systems.

A good overall estimator for the stiffness of the hydrogen bond network of an aqueous system is the reorientation dynamics of the water molecules. We address this property via the vector autocorrelation function of the dipole vector of the ensemble of water molecules in our different systems: all water molecules of the simulation cell are taken into account in this analysis to avoid any ambiguity of a distance cutoff. The set of autocorrelation functions of the solvated salt bridge with and without additional salt ions is shown in Fig. 2. The decay for pure water (i.e. no additional solvated ions beyond the Arg^+^-Asp^−^ pair) is linear, representing a mono-exponential shape (note the logarithmic scale of the plot). This shows that only a single type of activated microscopic process is involved, which is expected for regular liquid water. As soon as further ions are added (all in $\text{1}\,\text{M}$ concentration), the autocorrelation decay of the water is slowed down, and the shape is no longer linear. This illustrates a considerable stiffening of the hydrogen bond network and the existence of at least one more activated process for a sizeable subset of water molecules. Note that only a certain percentage of water molecules is affected to yield the observed slowing down. It is also remarkable that all salt combinations exhibit virtually the same autocorrelation decay, pointing to a quite unspecific effect. The time constant of the exponentially decaying autocorrelation function at long correlation times (~100 ps) is about five times smaller than the time constant for pure water at shorter times, meaning that the reorientation rate of the water molecules affected by the solvated ions is significantly smaller than that of pure water. We have performed a similar analysis of the autocorrelation functions of the OH bond vectors, with similar results (see the supporting information for details).

### Ion pair dissociation dynamics

Complementarily to the reorientation dynamics of the water, we determined directly the lifetimes of the ion–ion and ion–water aggregates. To this purpose, we computed the pairwise aggregate autocorrelation functions corresponding to each possible dimer (out of Arg^+^–A^−^/H_2_O, Asp^−^–Me^+^/H_2_O) via a suitable geometric criterion (see Methods section for details). Specifically, a good measure for such a statistically averaged lifetime is the total integral over the correlation function, which equals the exponential time constant in case of a mono-exponential decay.

We have summarized the resulting lifetimes for the most relevant ion-ion and ion-water dimers as function of the co-solvents in Table 1. The distribution of our computed values ranges from 30 ps to almost 5 ns, with a strong dependence on the nature of the involved ion and solvent composition. The most striking specific feature is the very long lifetime of the Li^+^ and also Na^+^ ions coordination to the Asp^−^ oxygen atoms, as opposed to all other combinations. This observation is consistent with the strongly kosmotropic nature of the Li^+^ ion, that is even more pronounced when its bonding partner is not neutral water but an anion. Nevertheless, the strength of the effect is surprising: the average lifetime is about fifty times longer than that of a water–Asp^−^ dimer.

**Table 1:** Intermittent lifetimes of complexes (in ps).

System	O (Asp)–H (Water)	H (Arg)–O (Water)	O (Asp)–Cation	H (Arg)–Anion	O (Water)–Cation	H (Water)–Anion
LiCl	85	63	4412	141	161	43
LiI	89	62	4729	91	152	31
NaCl	89	62	591	159	117	42
NaI	84	55	689	85	116	31
Pure water	64	39	—	—	—	—

Exchanging chloride against iodide results in a gradually weaker aggregation with both Arg^+^ and water molecules, which is consistent with the more chaotropic character of iodide. Note also the consistently stronger aggregation of the Cl^−^/I^−^ anions with the cationic sites of the amino acids, compared to the aggregation of Cl^−^/I^−^ with water molecules. This trend can be explained by the difference of a full elementary charge at Arg^+^ versus the dipole of the water molecules.

An interesting yet relatively small feature is that an unspecific effect is observed for water–Asp^−^/Arg^+^ hydrogen bonding: the presence of any type of additive ions in the solvent increases the stability of both water–Arg^+^ and water–Asp^−^ complexes by about 50%. This effect is stronger than expected, since the dissolved ions are not directly involved in the considered aggregates.

Our analysis of the dissociation dynamics of ion–ion and water–ion pairs thus illustrates the highly specific effect that solvated cations (especially Li^+^) have on the lifetime of complexes that they form with the Asp^−^ site; in turn, the lifetimes of the other possible complexes remain only modestly affected.

### Coordination strength of the salt bridge

**Figure 3:**
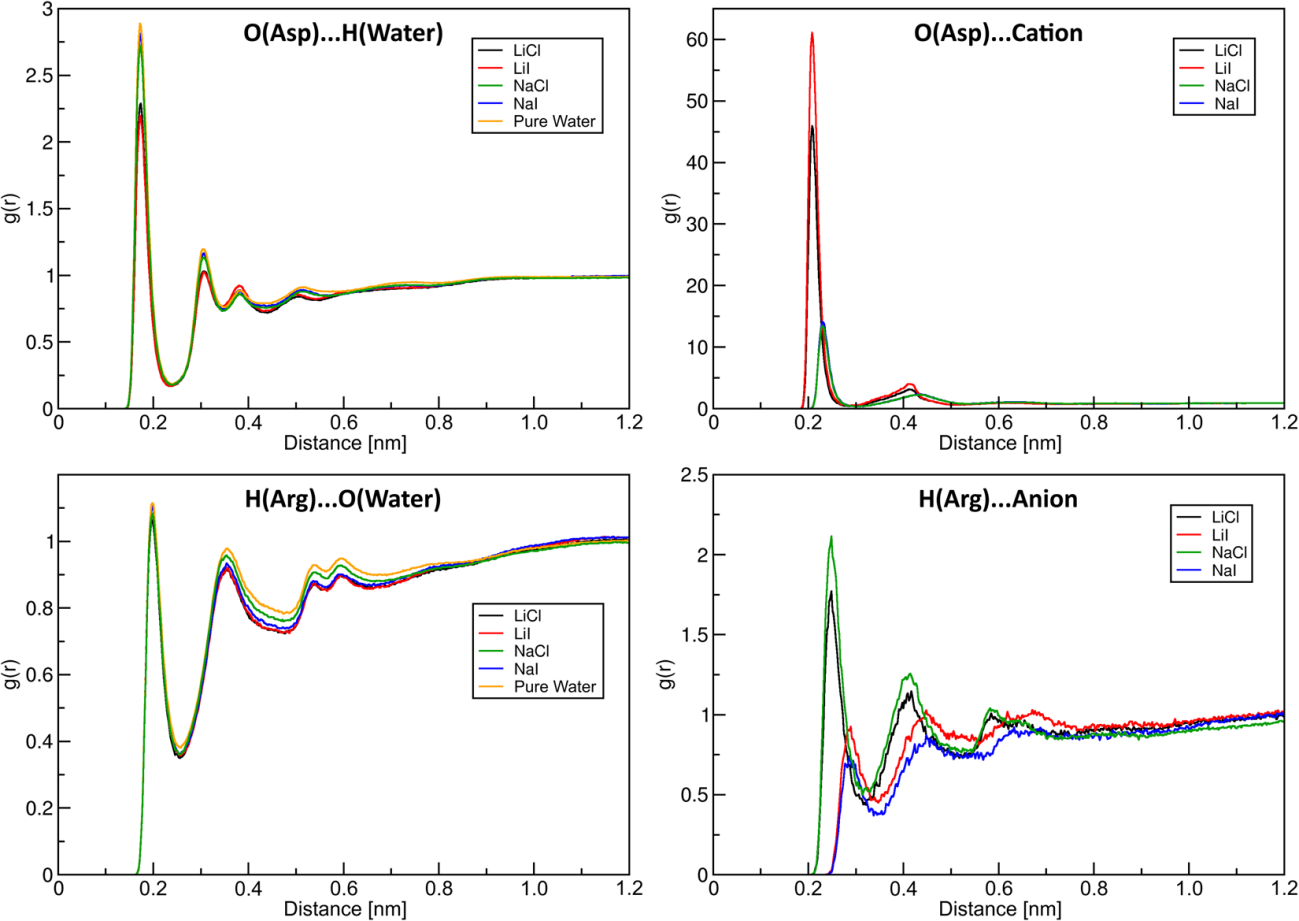
Radial distribution functions (RDF) between ion–ion and ion–water, analysis performed over all length of a corresponding trajectory.

The significantly prolonged lifetime of complexes between the Li^+^ ions and the anionic amino acid site raises the question about the statistical relevance of this aggregate, i.e. how persistent such a Asp^−^-Li^+^ coordination is compared to the competing Asp^−^-water coordination and the complementary Arg^+^-Cl^−^/I^−^/water coordination complexes. To this purpose, we have computed the radial distribution functions (RDFs) of the corresponding sites, shown in Fig. 3.

We observe a striking difference in the coordination of the Asp^−^ with Li^+^/Na^+^ and water. The first peak in the Li^+^ RDFs is about twenty times higher than that of water, which correlates well with the findings related to the corresponding aggregate lifetimes. It should be noted though that one of the reasons this difference appears large is due to the normalization of the RDF to the overall concentration (which is lower for Li^+^ and Na^+^ ions than it is for H_2_O molecules). Upon integration of the first peak in the RDF, it turns out that an Asp^−^-Li^+^ complex exists for about 50% of the time within our trajectory. This means that the interaction is enthalpically so favorable that it compensates the relatively low concentration of Li^+^ ions (compared to water molecules), resulting in an almost persistent structural feature. Note that the level of theory of our molecular dynamics simulation is not ideally suited for such strong interactions at the edge to a semi-covalent bond, a more accurate choice would be an ab initio based MD (presently ongoing). Nevertheless, the difference between Li^+^ and water coordination to Asp^−^ is very significant. Again, the Asp^−^-Na^+^ complex is much less prominent, which is consistent with the more bulky character of the sodium ion.

These findings correlate well with the average statistical ion density between the salt bridge charge groups, as illustrated in Figure S-18 (see the Supporting Information).The anions are generally repelled from the “salt bridge region” between Asp^−^ and Arg^+^, with the effect being much stronger for iodide than for chloride. For the cations, it differs. Lithium seems to be attracted to that region, with density ratios larger than 1. Sodium, however, seems to be slightly repelled, with density ratios slightly below 1.

The coordination of the cationic amino acid to the anions turns out to be less spectacular. While the Arg^+^-Cl^−^ peak in the RDF in Fig. 3 is more than twice as strong as the Arg^+^-I^−^ counterpart, there is no visible effect of the solvated salt on the Arg^+^-water coordination. The integration of the first peak in the Arg^+^-Cl^−^ and Arg^+^-I^−^ radial distribution functions yields a low degree of persistence for the ionic coordination: an Arg^+^-Cl^−^ ion pair exists in only about 6% of the simulated time, and the probability for an Arg^+^-I^−^ complex is around 4%.

### Mean force along salt bridge dissociation

**Figure 4:**
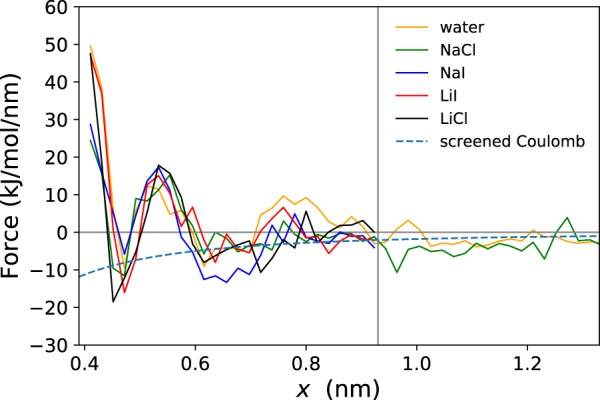
Mean force as a function of distance between guanidinium carbon - carboxylic carbon of the Arg-Asp salt bridge, together with screened Coulomb force (*ε*_*r*_ = 80).

We have computed the mean force between the anionic and cationic sites of the salt bridge in the different solutions during a forced dissociation event. The intermolecular force curve as a function of the distance between the carboxylic and guanidinium groups (i.e. the centers of charge) is shown in Fig. 4, along with the analytic screened Coulomb attraction between two elementary charges assuming a dielectric constant of *ε*_*r*_ = 80. The force curve exhibits a repulsive nature for distances below the equilibrium value of about *x* = 0.43 nm, followed by an attractive regime in the range 0.43 nm ≤ *x* ≤ 0.5 nm and again a repulsive window at 0.5 nm ≤ *x* ≤ 0.6 nm. Beyond that distance, the force approximately follows a screened Coulomb attraction. While the magnitude of the inter-ionic forces is relatively small compared to the considerable noise, the coinciding positions for the zeros of the force (i.e. equilibrium distances) increase the reliability of the ranges corresponding to attractive and repulsive regimes. It should be noted that the force curves for different salt additives do not exhibit any systematic differences above the noise level.

First, it is surprising that the width of attractive range (first minimum in Fig. 4) around *x* = 0.46 nm is comparable to the subsequent repulsive window (first maximum in Fig. 4). The magnitudes of the attractive/repulsive forces are about the same. Both observations indicate that there is actually little to no energetic preference for an intact salt bridge in solution, there is even a considerable energy barrier for its formation. Equally surprising is the missing dependence on either ion species for the additional salt in the solution. The very pronounced structural and dynamical features induced by the Li^+^ and Na^+^ ions have no visible effect on the energetic level. While the absence of any specific or unspecific effect can be explained for the anion (Cl^−^ and I^−^) via the comparably weak complex formation preference, it is not obvious why a quasi persistent Li^+^ coordination of the Asp^−^ oxygen atoms does not affect the inter-molecular forces on the amino acid residues. To a certain extent, geometric considerations based on the spatial distribution functions (see the supporting information) can help to understand this phenomenon: early during our simulations, Asp^−^ rotates so that only one of its carboxyl oxygen atoms is hydrogen-bonded to the Arg^+^ guanidinium protons, leaving the remaining carboxyl oxygen atom free to bind to a Li^+^.

## Discussion

We have investigated the influence of external constraints on a protein salt bridge, specifically the effect of simple salts on an Arginine–Aspartic acid dimer in aqueous solution, by means of molecular dynamics simulations. Besides structural and dynamical features at equilibrium, we have computed the mean force along the dissociation pathway of the salt bridge.

The analysis of our MD trajectories reveals a number of significant consequences of the presence of additional ions in the solution. The hydrogen bonding network of water becomes more rigid, reflected in the emergence of a slower rotational relaxation process (see Fig. 2): a closer analysis reveals that only those water molecules in direct contact with ions are affected. Such a behaviour was observed earlier experimentally by NMR measurements, where different clusters of solvatomers manifested themselves by an offset of the average chemical shift of water protons, as well as by a slowing down of their relaxation time^18,23,24^. However, these effects remain local to the first solvation sphere of the ions and do not significantly change the bulk properties of the system^28^. The same picture emerges for the different sets of ion pairs (Asp^−^–Li^+^/Na^+^/water, Arg^+^–Cl^−^/I^−^/water): more kosmotropic ions Li^+^ and Cl^−^ form longer-living complexes compared to more chaotropic Na^+^ and I^−^. Especially, the complex Li^+^–Asp^−^ is strikingly stable and persisted for ~50% of simulation time. Neither ion pair, however, induces structural or dynamical changes beyond its immediate vicinity. One can extrapolate these results to higher concentrations: with more ions added to the solution more and more water molecules will be participating in building up their solvation shells. This in turn will eventually lead to a slowing down of diffusion processes, indicating the formation of ‘stable’ water–ion aggregates, as was found in simulations of 9 M LiI aqueous solution^24^.

In contrast to these findings, which are in agreement with both chemical intuition and literature, the shape of the intermolecular force along the dissociation coordinate of the salt bridge indicates that the commonly assumed function of a protein salt bridge as a structural driving force may need to be revised^5^. The weakness of the attractive interaction at equilibrium distance combined with the presence of the repulsive force of a similar magnitude at 0.55 nm illustrates that the thermodynamic relevance of a salt bridge in such environment is much smaller than normally assumed.

To summarize, we have shown that Hofmeister ions influence the behavior of a solvated salt bridge in a very local and specific manner, namely the formation of tight ionic pairs between Li^+^/Na^+^ and Asp^−^. Bulk properties, however, are almost unaffected. Moreover, our findings show that the enthalpic function of the solvated salt bridge is minor, regardless of the presence of solvated ions. Specifically, the Li^+^–Asp^−^ interaction clearly calls for a more in-depth investigation that should be carried out using ab initio molecular dynamics simulations that are able to capture the range of hydrogen bonding versus covalent/coordination bonding at a very high level of accuracy.

## Methods

Classical Molecular Dynamic simulations were performed in GROMACS 2016.3^36,37^. We have used the AMBER03ws force field together with the TIP4PEw water model^29^. Ion parameters were taken from Gee *et al*.^35^. We have used a CSVR thermostat^38^ to keep 296 K temperature of the system together with Parrinello–Rahman barostat^39,40^ with *τ*_*p*_ = 1 ps. We have simulated a capped arginine-glutamate salt bridge^29^ in side-on conformation in 6/4/4 nm periodic box. The same setup was used in a paper by Chong *et al*.^29^, where authors have benchmarked performance of different force-fields as well as different water models. The structure was first equilibrated, solvated and optimized in pure water. Then ions were added into the simulation cell. In our simulation we have used LiI, LiCl, NaI, NaCl with 1 M concentration (58 anions and 58 cations). Same starting geometries of the salt bridge were used as starting points for all salts. Distance between charge centers of two amino acids, namely carboxylic carbon of Asp^−^ and guanidinium carbon of Arg^+^, was chosen as a reaction coordinate for cz trajectories (discussed in the main article), as well as distance between the C_*α*_ carbons for ca trajectories (see Supporting Information). To check reproducibility, we have repeated a simulation for NaCl changing the simulation temperature by 0.1 K, labeled NaClcheck. The routine of a simulation was: energy minimization followed by 3 ns equilibration of solvent. Umbrella pulling trajectory was performed with pull speed 0.0002 nm/ns for 270 ns for all salts, except for NaCl (cz and ca), NaClcheck (cz) and water(cz and ca) where the simulation was ran for 600 ns. To summarize, we have performed a forced dissociation of the Arginine–Aspartic acid anion dimer by applying a harmonic constraint to the reaction coordinate, and then moving the minimum of the applied potential along the coordinate with a constant velocity: ‘Umbrella pulling’. Very slow pulling rate allowed us to sample all other degrees of freedom of the system in a single trajectory. The mean force was projected onto the reaction coordinate for every timestep.

Analysis of trajectories was performed with VMD^41^. The RDFs, vector autocorrelation plots and lifetimes were computed with the TRAVIS program package^42^. The plots within this article have been created with Pyplot library in Python as well as with xmgrace^43^.

A well-established way of investigating lifetimes of aggregates in a molecular dynamics trajectory is to define a geometric aggregation criterion and to autocorrelate the corresponding pair definition function for each possible bonding aggregate. The integral over the resulting autocorrelation function (ACF) can then be considered as the aggregate’s lifetime. In this work, we use a simple distance criterion to define aggregates between aspartate, arginine, anions, cations, and water. The distance cutoff values were derived from the position of the first minimum in the corresponding RDFs. We selected 300 pm for the Cation–O(Asp) distance; 330 pm for the Anion–H(Arg), Anion–H(Water), and Cation–O(Water) distance; 250 pm for the H(Water)–O(Asp) and O(Water)–H(Arg) distance. Lifetimes have been computed by using the intermittent formalism from Rapaport^44^, which allows aggregates to break and reform at a later time. These intermittent ACFs are much less prone to noise than the corresponding continuous functions, which do not permit for re-formation of aggregates. When considering intermittent ACFs, special care has to be taken in the case of finite–size systems. The functions will not fall to zero after very long correlation time, but to the *ensemble average* instead, which is the average probability of observing a hydrogen bond in one specific pair after very long simulation. This effect can be corrected by subtracting the ensemble average from the intermittent ACFs, which is called *ensemble average correction*. This correction was described in literature before^45^ and is applied here.

The radial distribution function curves are normalized to uniform particle density, which means that a value of 1 depicts a probability of finding a certain particle in a certain distance which equals the probability of finding that particle at any random point in the simulation cell.

## Electronic supplementary material

Supporting Information
