# Supplementary material for: Salt Bridge in Aqueous Solution: Strong Structural Motifs but Weak Enthalpic Effect
Source: Sci Rep. 2018 Sep 11;8:13626. doi: 10.1038/s41598-018-31935-z (PMC6133928; doi:10.1038/s41598-018-31935-z)
Supplement: Supplementary file 1 — Supporting Information [file 41598_2018_31935_MOESM1_ESM.pdf]

Salt Bridge in Aqueous Solution: Strong Structural Motifs but Weak Enthalpic Effect. Supporting Information

Svetlana Pylaeva[1], Martin Brehm[1], and Daniel Sebastiani[1,*]

[1]Martin Luther University Halle–Wittenberg, Institute for Physical Chemistry, 06120 Halle (Saale), Germany
[*]daniel.sebastiani@chemie.uni-halle.de

ABSTRACT

Salt bridges are elementary motifs of protein secondary and tertiary structure and are commonly associated with structural driving force that increases stability. Often found on the interface to the solvent, they are highly susceptible to solvent–solute interactions: primarily with water but also other cosolvents and ions. We have investigated the interplay of an Arginine–Aspartic acid salt bridge with simple salt ions in aqueous solution by means of molecular dynamics simulations. Besides structural and dynamical features at equilibrium, we have computed the mean force along the dissociation pathway of the salt bridge. We demonstrate that solvated ions influence the behavior of the salt bridge in a very specific and local way, namely the formation of tight ionic pairs Li^+/Na^+–Asp^-. Moreover, our findings show that the enthalpic relevance of the salt bridge is minor, regardless of the presence of solvated ions.

Table 1. Intermittent lifetimes of configurations (in ps).

System	O(Asp)–H(Water)	H(Arg)–O(Water)	O(Asp)–Cation	H(Arg)–Anion	O(Water)–Cation	H(Water)–Anion
LiCl_cz	85	63	4412	141	161	43
LiI_cz	89	62	4729	91	152	31
NaCl_cz	77	52	647	156	117	43
NaI_cz	84	55	689	85	116	31
Water_cz	64	39	-	-	-	-
Water_ca	57	36	-	-	-	-
NaCl_ca	69	48	624	153	117	43
NaClcheck_cz	89	62	591	159	117	42

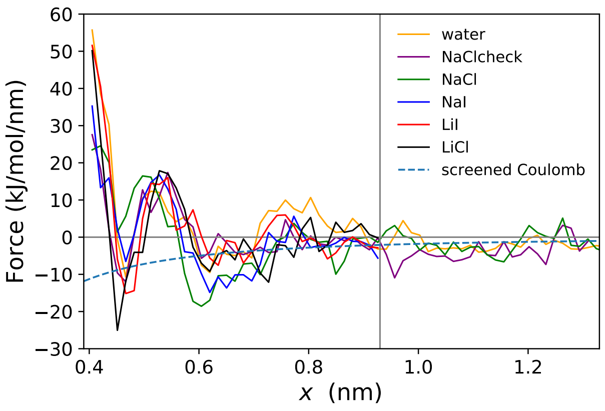

Figure 1. Mean force for all systems pulled along reaction coordinate connecting two centers of charge.

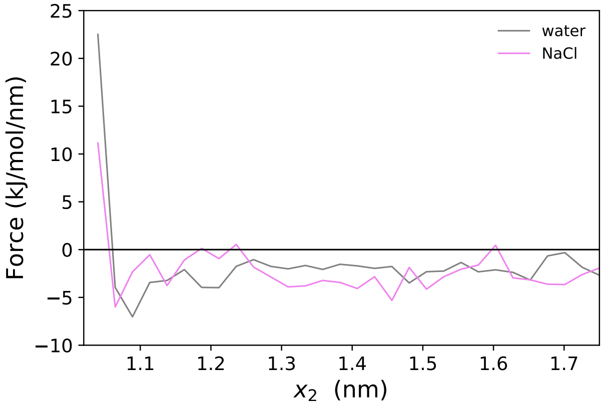

Figure 2. Mean force for all systems pulled along reaction coordinate connecting two C_α carbons.

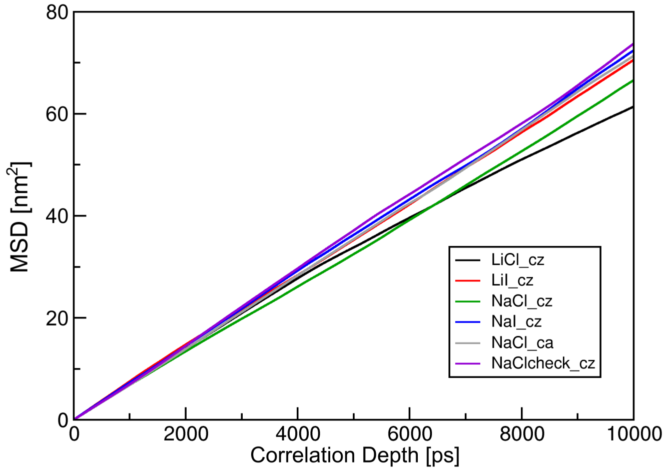

Figure 3. Mean square displacement (MSD) plots of the anions.

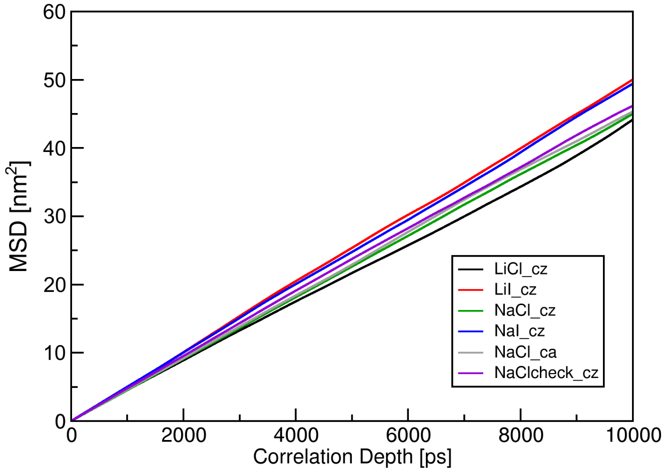

Figure 4. Mean square displacement (MSD) plots of the cations.

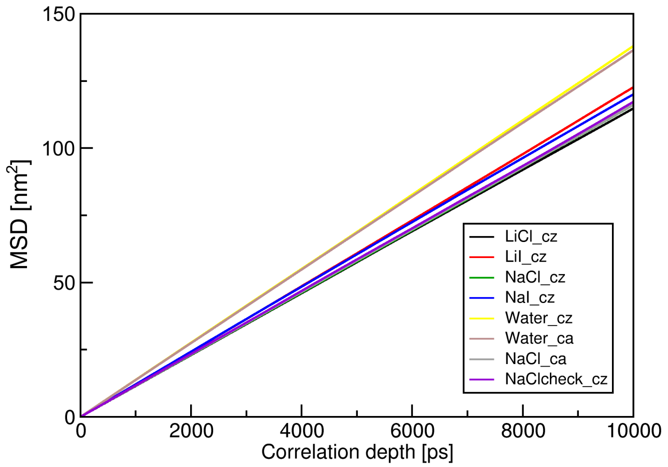

Figure 5. Mean square displacement (MSD) plots of the water molecule's center of mass.

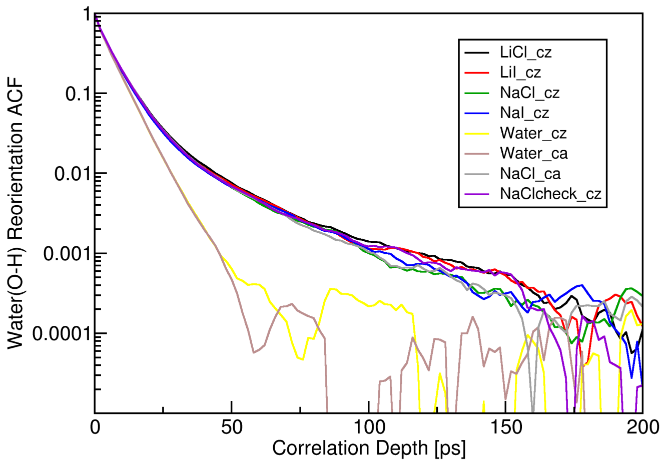

Figure 6. Vector autocorrelation functions of the O–H bond vector in water.

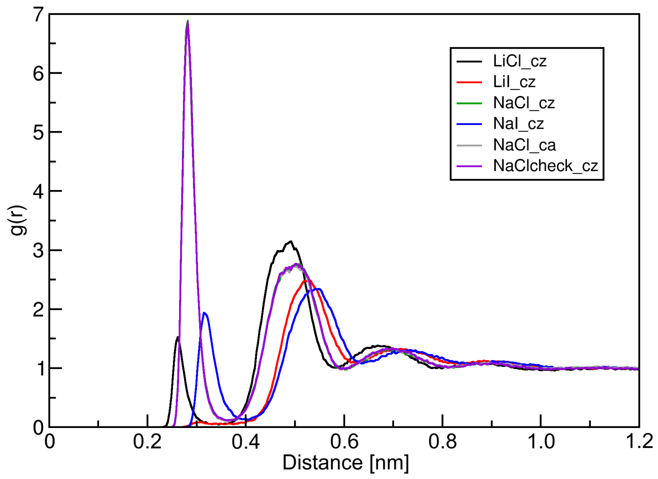

Figure 7. Radial distribution function (RDF) between anion and cation.

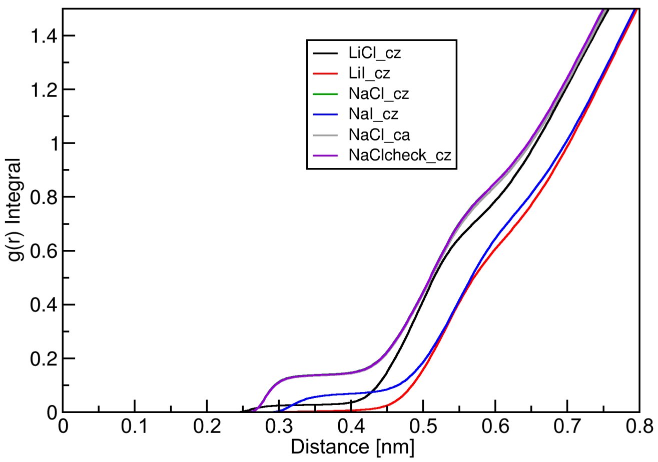

Figure 8. Number integrals of RDFs between anion and cation.

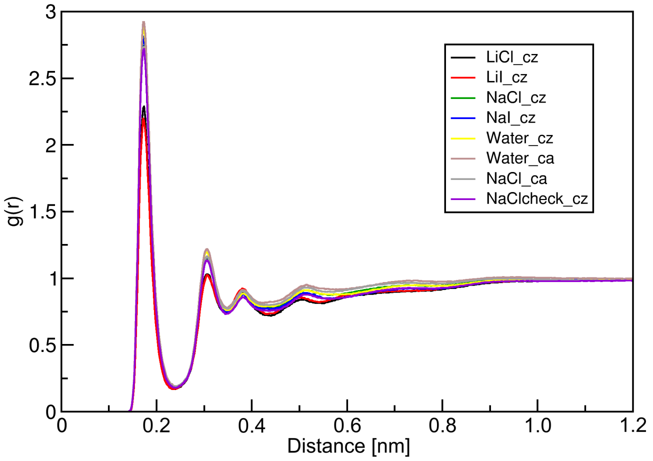

Figure 9. Radial distribution function (RDF) between O(Asp) and H(Water).

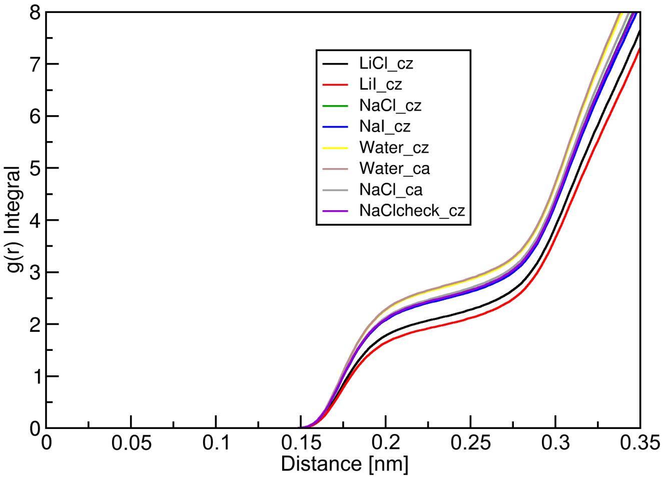

Figure 10. Number integrals of RDFs between O(Asp) and H(Water).

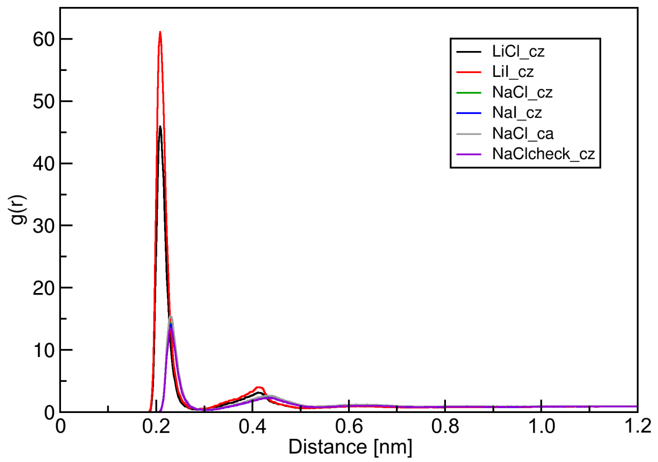

Figure 11. Radial distribution function (RDF) between O(Asp) and cation.

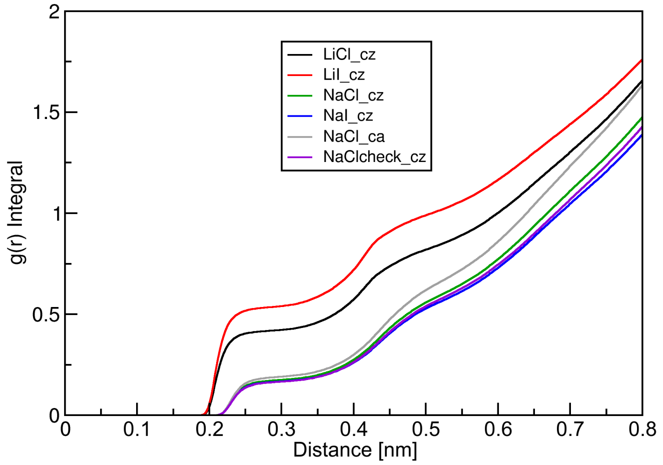

Figure 12. Number integrals of RDFs between O(Asp) and cation.

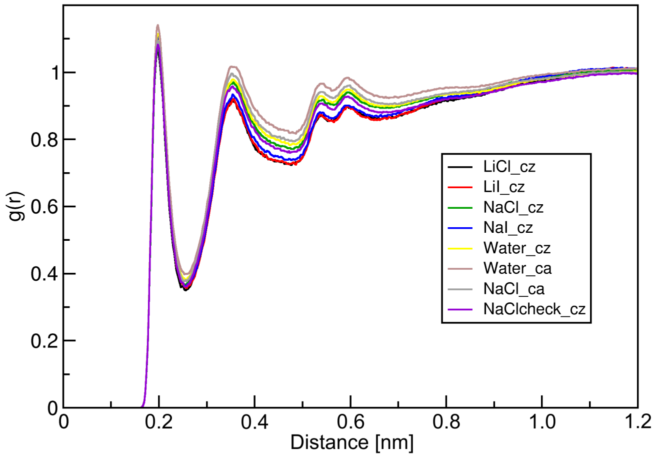

Figure 13. Radial distribution function (RDF) between H(Arg) and O(Water).

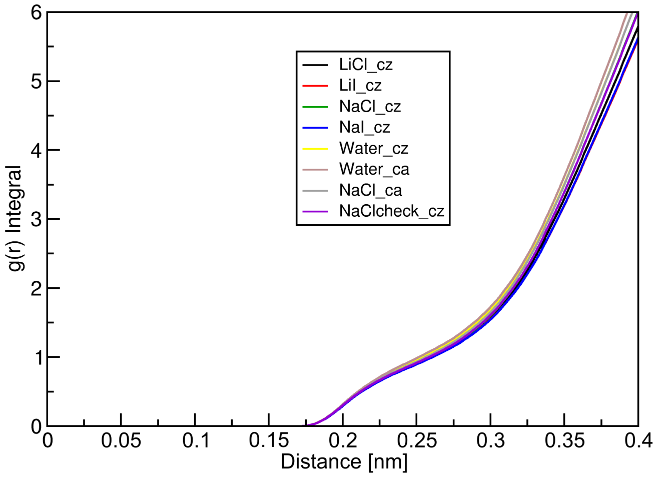

Figure 14. Number integrals of RDFs between H(Arg) and O(Water).

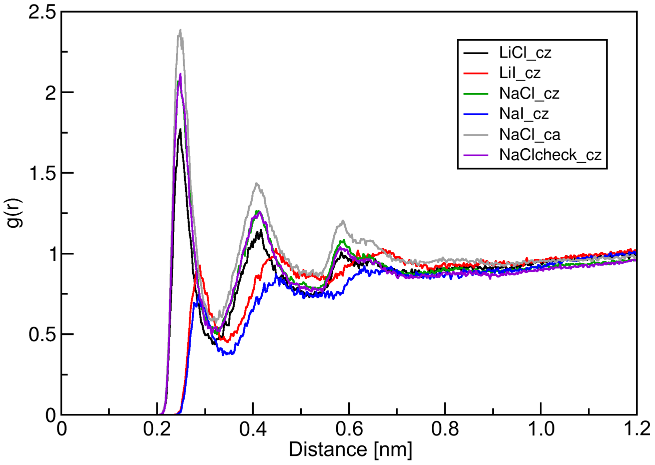

Figure 15. Radial distribution function (RDF) between H(Arg) and anion.

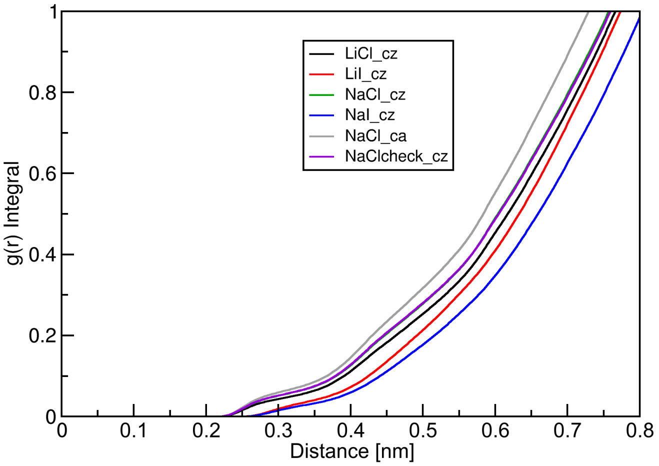

Figure 16. Number integrals of RDFs between H(Arg) and anion.

For an analysis presented in Figure 18 we first define a line between the charge centers of Asp^{-} and Arg^{+}. Around this line, we then define a cylinder with a fixed diameter. The cylinder is not fixed, it moves together with Asp^{-} and Arg^{+}, and therefore always covers the salt bridge. We chose the diameter to be 0.8 nm, that is sufficient to cover all the hydrogen bond donors of arginine. Then, we count the number of anions/cations inside this cylinder in each time step of the trajectory. Together with the known (varying) volume of the cylinder, this gives us the concentration of ions within the cylinder. We then compute the ratio between concentration of ions within the cylinder and concentration in bulk, i.e., the excess of ions in the cylinder when compared to the bulk.

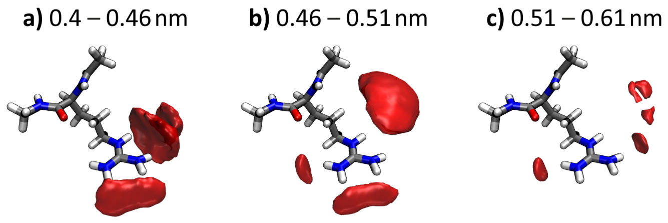

Figure 17. Spatial distribution function of Asp carboxylic oxygen atoms relative to Arg guanidinium group for different parts of mean force curve for pure water system.

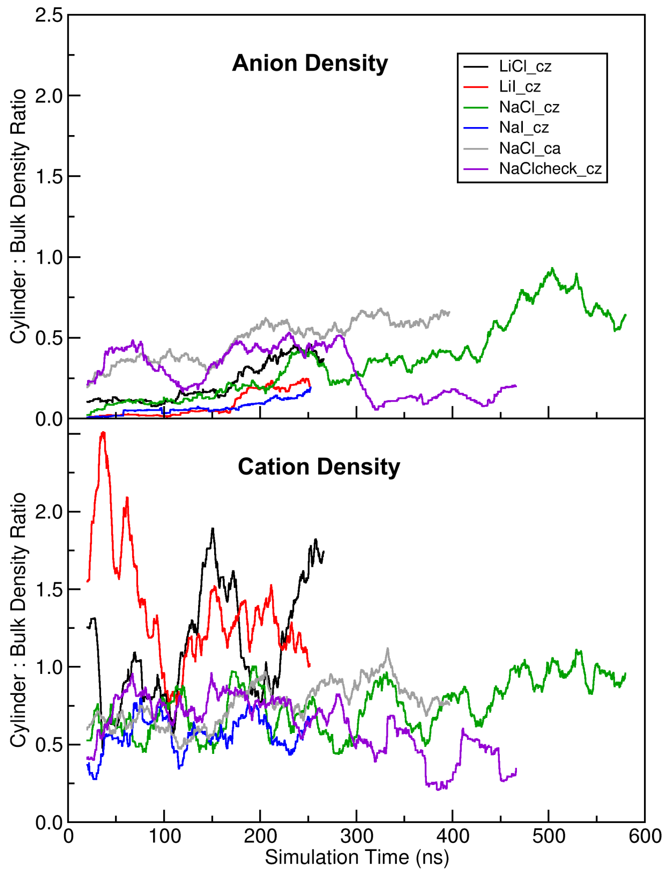

Figure 18. Ratio between concentration of ions in the vicinity of charge centers of the salt bridge and in bulk. See text for details.

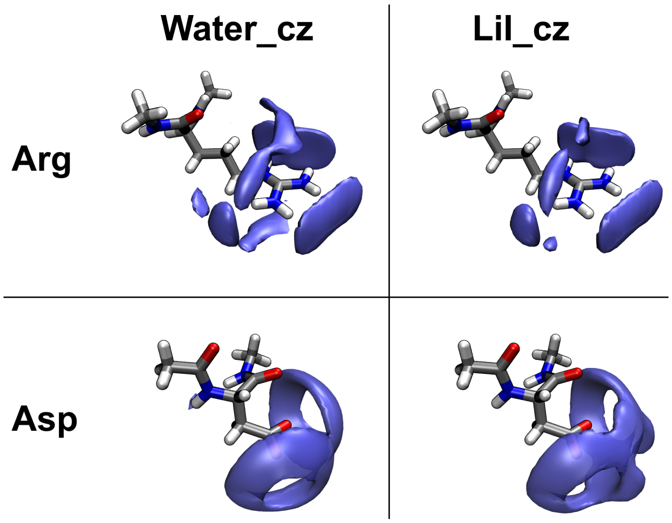

Figure 19. Spatial distribution functions of water center of mass around arginine (upper part) and aspartate (lower part) in the Water_cz trajectory (left part) and LiI_cz trajectory (right part). Surfaces are shown for an iso-value of 60 nm^{-3}. The first 100 ns of the trajectories were excluded from the analysis.
